# Activation of Polypropylene (PP) Fiber Surface with 1-Vinyl-1,2,4-triazole and Vinyl Acetate: Synthesis, Characterization, and Application in Cementitious Systems

**DOI:** 10.3390/ma18051071

**Published:** 2025-02-27

**Authors:** Yahya Kaya, Petek Balcı, Süleyman Özen, Ali Mardani, Ali Kara

**Affiliations:** 1 Department of Civil Engineering, Faculty of Engineering, Bursa Uludag University, 16059 Bursa, Turkey; 512126007@ogr.uludag.edu.tr; 2 Department of Polymer Materials, Faculty of Arts and Sciences, Bursa Uludağ University, 16059 Bursa, Turkey; petekbalci@outlook.com (P.B.); akara@uludag.edu.tr (A.K.); 3 Department of Civil Engineering, Faculty of Engineering and Natural Sciences, Bursa Technical University, 16059 Bursa, Turkey; suleyman.ozen@btu.edu.tr

**Keywords:** 1-Vinyl-1,2,4-Triazole, Vinyl Acetate, polypropylene fiber, coating, cementitious system

## Abstract

Recently, the potential of recycled materials to improve the performance of concrete and other building materials has become an important research topic. It is known that various methods are applied to improve the tensile strength and energy absorption capacity of cementitious systems. One of the most common of these methods is the addition of fibers to the mixture. In this study, the effects of surface-modified polypropylene (PP) fibers obtained from recycled masks on the mechanical properties of mortar mixtures were investigated. In order to improve the matrix–fiber interface performance, 6 mm and 12 mm long recycled PP fibers were chemically coated within the scope of surface modification using 1-Vinyl-1,2,4-Triazole and Vinyl Acetate. With this modification made on the surface of PP fibers, we aimed to increase the surface roughness of the fibers and improve their adhesion to the matrix. Thus, we aimed to increase the mechanical properties of mortar mixtures as a result of the fibers performing more effectively in the concrete matrix. FTIR AND SEM-EDS analyses confirmed the success of the modification and the applicability of 1-Vinyl-1,2,4-Triazole and Vinyl Acetate to the fiber surface and showed that the fibers were successfully modified. It is seen that the fibers modified with Vinyl Acetate exhibit superior performance in terms of both the workability and strength performance of cementitious systems compared to the fibers modified with 1-Vinyl-1,2,4-Triazole. This study provides a significant contribution to sustainable construction materials by revealing the potential of using recycled materials in cementitious systems.

## 1. Introduction

In recent years, extensive research has focused on various additives to enhance the performance of cementitious systems and broaden their potential applications [1]. In this regard, fiber-reinforced concretes have emerged as composite materials that significantly enhance the key properties of traditional concretes, including impact resistance, ductility, toughness, and crack mitigation. The incorporation of fibers not only improves the mechanical properties of cementitious systems but also contributes to the advancement of concrete technology by enhancing their durability [2,3].

Fibers play a crucial role in preventing crack propagation by absorbing stresses within the cement paste and transferring these stresses to the more resilient regions of the matrix [4]. The inclusion of fibers in concrete can exert both positive and negative effects on compressive strength; however, it significantly enhances impact strength by improving ductility and toughness. The increase in impact strength can primarily be attributed to the fibers’ ability to absorb impact energy and their role in the initiation and propagation of cracks [5,6]. A wide range of fibers, such as polypropylene, glass, steel, basalt, polyvinyl alcohol, polyamide, ceramics, polyethylene, kevlar, nylon, and natural fibers, can be incorporated into concrete mixes. Among these, polypropylene and steel are the most commonly used fiber types. In addition to fiber type, factors such as fiber slenderness, dosage, and shape also significantly influence the impact strength of concrete [7].

The historical development of concrete reveals that some of the earliest examples of fiber-reinforced composites include baked clay and mortars reinforced with straw and animal hair. In contemporary concrete mixtures, fibers are generally categorized into natural and synthetic types. Synthetic fibers are primarily derived from chemical materials, with steel, glass, carbon, and polypropylene being the most commonly used in the concrete industry. However, the production of synthetic fibers contributes significantly to greenhouse gas emissions, leading to a growing demand for natural fibers and recycled waste fibers. Despite this shift, natural and recycled fibers have various limitations, which restrict their widespread use.

The mechanical and durability properties of fiber-reinforced cementitious systems are largely influenced by the strength of the interfacial interaction between the fiber and the matrix. Polypropylene (PP) fibers, due to their chemical inertness, typically exhibit poor adhesion to the cement matrix. To enhance the performance of these fibers, it is crucial to improve the interaction between the fiber and the cement paste. Numerous studies have focused on strengthening this interaction, with one of the most common methods being the modification of fiber shape and surface roughness through mechanical treatments. For instance, incorporating hooks or twisting the fibers are effective strategies for enhancing the fiber–matrix bond strength. While these modifications can significantly improve the interaction, they may also reduce the fibers’ stiffness. Additionally, techniques such as hooking may present practical challenges when applied to microfibers.

In addition to physical modifications, chemical treatments are often employed to increase the compatibility between the fiber and the matrix interface. Fibers are generally classified as hydrophilic or hydrophobic. Hydrophilic fibers, which contain polar groups such as hydroxyl or carboxyl groups in their polymer chains, can form ionic bonds with Ca2+ ions in cementitious systems or covalent bonds with calcium silicate hydrate (C-S-H). This enables hydrophilic fibers to establish a chemical bond with the cement while also enhancing physical durability through surface friction. On the other hand, hydrophobic fibers lack polar groups, preventing the formation of chemical bonds with cement. As a result, the interaction at the cement–fiber interface is governed solely by frictional forces. Similarly, synthetic fibers like polypropylene (PP), which are devoid of polar groups, exhibit cement–fiber interactions that rely exclusively on friction.

Surface modifications are commonly employed to enhance the hydrophilicity and chemical reactivity of synthetic fibers. These modifications typically involve introducing polar groups, often oxygen-containing, onto the fiber surface. Two principal approaches are used to chemically incorporate polar groups or nanomaterials onto synthetic fibers. The first method includes oxidation processes, such as cold plasma treatment or ozonation [8]. The second approach involves applying coatings of materials like carbon nanofibers, polydopamine, graphene oxide [9], SiO_2_ nanoparticles [10], and CaCO_3_ nanoparticles [11] onto the fiber surface.

Research on improving the performance of polypropylene (PP) fibers within cement matrices has yielded valuable insights into enhancing fiber–matrix interactions. Existing studies predominantly focus on increasing surface polarity through various modification techniques. However, limited attention has been given to the modification of recycled PP fibers to simultaneously enhance their recovery and performance. This study addresses this gap by performing chemical surface modification on 6 mm and 12 mm recycled PP fibers sourced from discarded masks to optimize cement–fiber interface performance. Compressive and tensile strengths of mortar samples incorporating 1-Vinyl-1,2,4-Triazole-modified PP fibers (VTPP) and Vinyl Acetate-modified PP fibers (VAPP) were evaluated at 7 and 28 days.

## 2. Materials and Methods

### 2.1. Materials

CEM 42.5 R-type cement was utilized as the binder in this study (Bursa Cement Factory Inc., Bursa, Türkiye). The chemical composition, along with the physical and mechanical properties of the cement, as provided by the manufacturer, are presented in Table 1.

**Figure 1:**
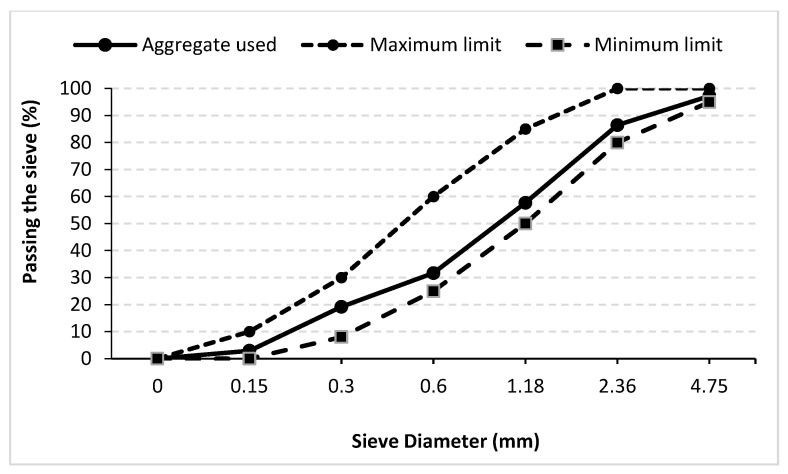
Gradation curve of the fine aggregate used compared to ASTM C33 standard limits.

Crushed limestone sand with a grain size range of 0–4 mm, a water absorption capacity of 2.03%, and a specific gravity of 2.6% was used in the production of mortar mixtures. The gradation curve of the aggregate and the ASTM C33 standard limits are shown in Figure 1.

A single type of polycarboxylate ether-based high-range water-reducing admixture (PCE) was utilized at varying dosages to achieve the desired flow values in the mortar mixtures (Polisan Chem Inc., Gebze, Türkiye). Key properties of the water-reducing admixture, as provided by the manufacturer, are listed in Table 2.

**Figure 2:**
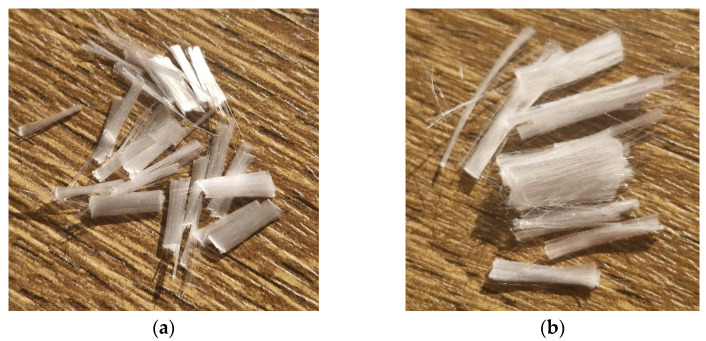
PP fibers of 6 mm (**a**) and 12 mm (**b**) in length.

The polypropylene (PP) fibers used in this study were produced by recycling waste masks through a specialized recycling process (Mert Recycling Factory, Gaziantep, Türkiye). The process involved sterilizing and melting the masks at high temperatures to produce PP granules, which were then pressed under heat to form recycled PP fibers. Fibers of 6 mm and 12 mm lengths were used separately (Figure 2). Key properties of the fibers, as provided by the manufacturer, are summarized in Table 3.

#### 2.1.1. Materials Used in the Synthesis of Activation of PP Polymer Surface with 1-Vinyl-1,2,4-Triazole

For the surface activation of PP fibers using 1-Vinyl-1,2,4-Triazole, the synthesis involved the following components: 4 g of recycled polypropylene fibers (6 mm and 12 mm lengths), 0.1 g of 1-Vinyl-1,2,4-Triazole (≥97.0%, Sigma-Aldrich, CAS No: 2764-83-2), 0.5 g of Potassium Persulfate (≥99.0%, Sigma-Aldrich, CAS No: 7727-21-1), 20 mL of distilled water, and 5 mL of ethanol (≥99.8%, CAS No: 64-17-5). These quantities were precisely prepared to perform the surface activation process. The resulting fibers, modified with 1-Vinyl-1,2,4-Triazole, were designated as VTPP.

#### 2.1.2. Materials Used in the Synthesis of PP Polymer Surface Activation with Vinyl Acetate

To activate the polypropylene (PP) polymer surface with Vinyl Acetate, 4 g of recycled polypropylene fibers (6 mm and 12 mm lengths), 0.1 g of Vinyl Acetate (≥99.8%, Sigma-Aldrich, CAS No: 108-05-4), 0.5 g of Potassium Persulfate (≥99.0%, Sigma-Aldrich, CAS No: 7727-21-1), 20 mL of distilled water, and 5 mL of ethanol (≥99.8%, Merck, CAS No: 64-17-5) were used in the process. These components were accurately measured and prepared for the surface activation procedure. The fibers modified by this method using Vinyl Acetate were designated as VAPP.

### 2.2. Methods

#### 2.2.1. Synthesis of Activation of PP Polymer Surface by 1-Vinyl-1,2,4-Triazole

A potassium persulfate radical initiator solution was introduced into a hot-water-jacketed glass reactor, where the polypropylene (PP) fibers were then added. The reactor was operated at 950 rpm and a temperature of 70 °C, allowing the activation of the PP fiber surfaces with 1-Vinyl-1,2,4-Triazole. The reaction was maintained at 70 °C for 5 h with magnetic stirring, followed by an additional 12 h at 25 °C, ensuring proper stabilization and preventing thermal degradation of the modified PP fibers. After the synthesis, the fibers were dried in an oven at 70 °C for 45 min. This method resulted in the coating of the PP fibers.

#### 2.2.2. Synthesis of Activation of PP Polymer Surface with Vinyl Acetate

Surface-activated polypropylene (PP) fibers were obtained by synthesizing the fiber surfaces with Vinyl Acetate in a hot-water-jacketed glass reactor, operating at 70 °C and 950 rpm, with the addition of a potassium persulfate radical initiator. The reaction proceeded at 70 °C for 5 h under magnetic stirring, then continued for 12 h at 25 °C to promote stabilization and minimize the risk of thermal degradation in the modified PP fibers. After the synthesis, the fibers were placed in an oven at 70 °C for 45 min to ensure proper drying. The surface-activated PP fibers obtained from the synthesis were characterized using FTIR and SEM-EDX techniques.

#### 2.2.3. PP Fiber Surface Modification

**Figure 3:**
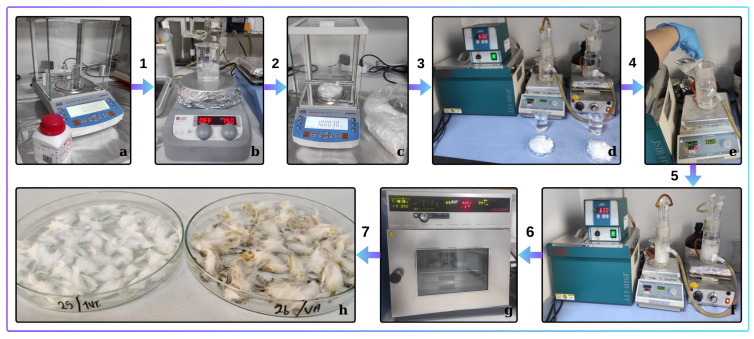
The synthesis steps, preparation of chemical solutions, surface activation, and drying processes for activating the PP fiber surface using 1-Vinyl-1,2,4-Triazole and Vinyl Acetate monomers are illustrated in the figure. (**a**) Weighing of chemicals. (**b**) Dissolution of chemicals on a magnetic stirrer. (**c**) Weighing of PP fibers. (**d**) Opening the hot water bath before the reaction. (**e**) Addition of pp fibers and chemicals into glass reactors, respectively. (**f**) Reaction start moment. (**g**) Drying stage of PP fibers in the oven. (**h**) Appearance of ready-to-use pp fibers after the drying stage.

PP fibers exhibit a smooth and flat surface structure with a waxy appearance. The strength of these fibers ranges from 13 to 30 cN/tex, providing a broad spectrum of performance across various applications. In comparison, high-density polyethylene (HDPE) fibers have a strength of 70–90 cN/tex, while low-density polyethylene (LDPE) fibers range from 15 to 60 cN/tex. These strength variations necessitate surface activation processes to enhance the mechanical properties of PP fibers, improving their performance in cementitious systems. In the synthesis process, solid chemicals were weighed (1) and dissolved in suitable solvents (2), while PP fibers were also weighed (3). Surface activation was carried out using 1-Vinyl-1,2,4-Triazole and Vinyl Acetate monomers in separate reactors at 70 °C and 950 rpm stirring speed under a fume hood with a magnetic stirrer (4, 5). The resulting solutions were decanted and dried in a vacuum oven at 70 °C (7, 8). The synthesis steps for activating the PP polymer surface with 1-Vinyl-1,2,4-Triazole and Vinyl Acetate are shown in Figure 3.

#### 2.2.4. Preparation of Mortar Mixtures

Mortar mix calculations were performed for a 1 dm^3^ volume, and the mixes were produced following the ASTM C109 standard. The water/binder ratio and flow were maintained constant at 0.485 and 200 ± 20 mm, respectively, for all mixtures. In addition to the fiber-free control mix, six batches of mortar were prepared using 6 mm and 12 mm long PP fibers, as well as modified PP fibers. In the fiber-containing mixtures, the fiber was substituted for the aggregate at 0.50% of the total volume. The chosen fiber ratio was based on preliminary studies conducted by the authors to determine the optimal ratio. The mixtures were named according to the fiber type and length. For instance, the mix with 12 mm long polypropylene fibers was labeled as 12-PP, the mix with 6 mm long PP fibers modified with 1-Vinyl-1,2,4-Triazole was named 6-VTPP, and the mix with 6 mm long PP fibers modified with Vinyl Acetate was named 6-VAPP. A water-reducing admixture was added at various dosages to achieve the desired flow. Seven mortar mixes were produced in total. The amounts of materials used in the production of 1 dm^3^ mortar mixtures and the corresponding flow values are presented in Table 4. The specimens were cured in lime-saturated water at a temperature of 20 °C until the test day.

#### 2.2.5. Mixture Experiments

Flow, compression, and flexural tests of mortar mixtures were determined in accordance with the ASTM C1437, EN 196-1, and EN 196-1 standards, respectively.

#### 2.2.6. Fiber Characterization Processes

##### Fourier Transform Infrared Spectroscopy (FTIR) Analysis

The chemical structures of the synthesized fibers were determined by FTIR analysis. FTIR spectra were obtained with a Perkin Elmer Spectrum Two FTIR spectrometer. The analysis was performed in the wavelength range between 4000 cm^−1^ and 400 cm^−1^ with a resolution of 4 cm^−1^ by performing a total of thirty-two scans.

##### Scanning Electron Microscopy (SEM-EDS) Analysis

Surface images of the synthesized fibers were obtained using a Zeiss/Gemini 300 microscope (ZEISS, Jena, Germany). The chemical compositions of the coating materials on the surface were analyzed by the EDS (Energy Dispersive Spectroscopy) method using a Bruker/XFlash 6100 device (Bruker Corporation, Billerica, MA, USA). These methods investigate the surface properties of the fibers and the chemical composition of the coating materials.

## 3. Results

### 3.1. Characterization of PP Polymer Surface Activation by 1-Vinyl-1,2,4-Triazole

**Figure 4:**
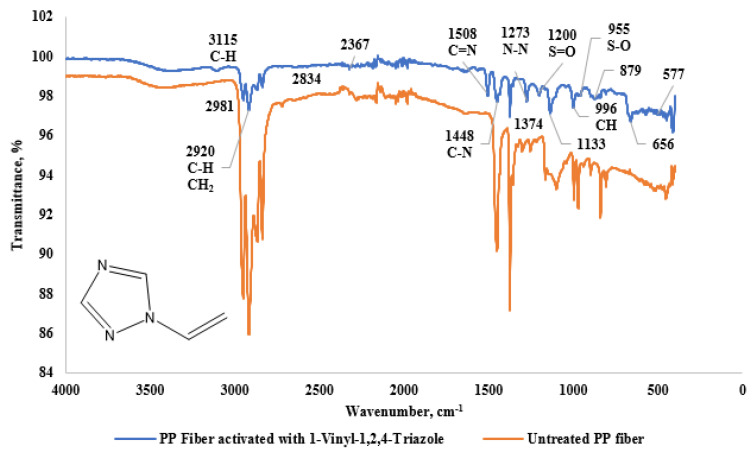
FTIR graph of PP fiber activated with 1-Vinyl-1,2,4-Triazole and untreated PP fiber.

The FTIR spectrum shown in Figure 4 compares untreated PP fibers with PP fibers modified using 1-Vinyl-1,2,4-Triazole. The spectrum for the modified fibers displays characteristic peaks corresponding to the triazole rings at 3115 cm^−1^ (C-H), 1508 cm^−1^ (C=N), 1448 cm^−1^ (C-N), 1273 cm^−1^ (N-N), 996 cm^−1^ (C-H), and 656 cm^−1^ (C-N), as identified by [12]. In addition, the peaks at 1133 cm^−1^ and 577 cm^−1^ in the fingerprint region correspond to C-H bonds in the 3-Chloro-2-Chloromethyl-1-Propene molecule [13]. Furthermore, peaks in the fingerprint region at 1200 cm^−1^, 955 cm^−1^, and 1219 cm^−1^ are attributed to S=O, S-O, and SO_4_^2−^ bonds, respectively, suggesting the presence of potassium persulfate.

The formation of C=N, C-N, and N-N peaks indicates that triazole rings are successfully attached to the fiber surface. The intensification of the C-H peak at 3115 cm^−1^ confirms the changes in the organic structure. FTIR results show that the modification is successful and the fiber surface becomes more hydrophilic. After modification with 1-Vinyl-1,2,4-Triazole, an increase in intensity was observed in the peaks at 1508 cm^−1^ (C=N) and 1448 cm^−1^ (C-N). This indicates that the triazole rings were successfully bonded to the pp fiber surface. In the study conducted by Pozdnyakov et al. (2017), it was stated that a significant increase was observed in the C=N and C-N peaks as a result of the interaction of 1-Vinyl-1,2,4-Triazole with the surface. In addition, an increase occurred in the N-N peak at 1273 cm^−1^, confirming that the triazole rings were integrated into the polymer matrix [12]. This modification may cause PP fibers to become more hydrophilic by increasing the surface energy. Similarly, Meltonyan et al. [14] stated that triazole derivatives increase the water retention capacity by adhering to the surface, which may affect the mechanical performance of the material.

**Figure 5:**
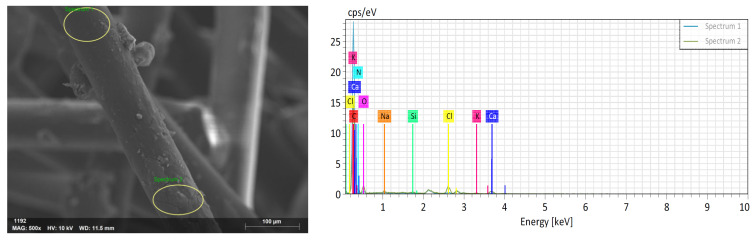
SEM image and EDS graph of PP fiber with 1-Vinyl-1,2,4-Triazole.

The SEM image and EDS analysis of the modified fiber are shown in Figure 5, and the corresponding normalized mass concentration (%) values of the PP fiber with 1-Vinyl-1,2,4-Triazole are provided in Table 5.

**Figure 6:**
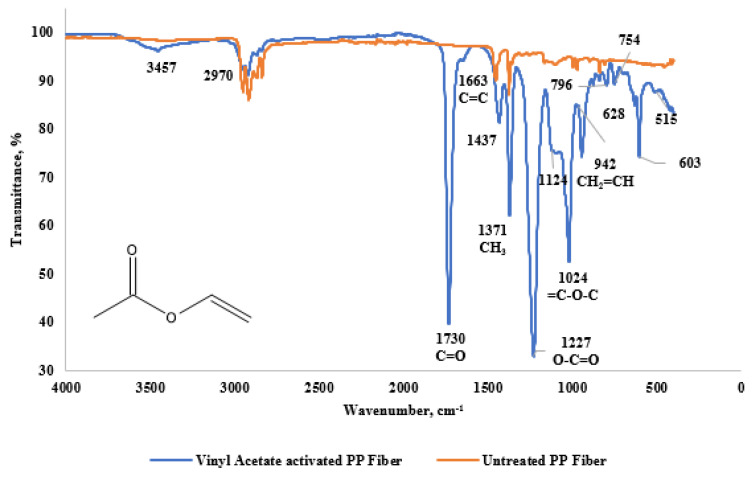
FTIR graph of PP fiber activated with Vinyl Acetate and untreated PP fiber.

The FTIR spectrum of Vinyl Acetate shown in Figure 6 displays characteristic bands that are associated with the vinyl group. The band at 1663 cm^−1^ corresponds to the stretching vibration of the C=C bond, while the band at 877 cm^−1^ is attributed to the C-H bending vibration. Additional key features include the stretching vibration at 942 cm^−1^ for C=C and the ester group at 1124 cm^−1^ (=C-O-C stretching vibration), which are indicative of Vinyl Acetate. Moreover, the C=O stretching vibrations at 1730 cm^−1^ and 1227 cm^−1^ are likely related to the ester group in Vinyl Acetate [15,16].

In the fingerprint region, the peaks at 1124 cm^−1^ and 515 cm^−1^ are associated with C-H bonds from the 3-Chloro-2-Chloromethyl-1-Propene molecule. Additionally, the 1227 cm^−1^ peak corresponds to S=O bonds, while 942 cm^−1^ is attributed to S-O bonds, and the 1219 cm^−1^ peak corresponds to SO_4_^2−^ bonds, suggesting the presence of potassium persulfate, as noted by Cherifi et al. [15].

The peaks at 1730 cm^−1^ (C=O) and 1124 cm^−1^ (C-O-C) prove that the ester structure is attached to the fiber surface. Modifying the fibers to make them more hydrophobic can optimize their interaction with the cement matrix. In the Vinyl Acetate modification, a significant increase was observed in the peak distributions at 1730 cm^−1^ (C=O) and 1124 cm^−1^ (C-O-C). This result indicates that esterification rates are present. Squillace et al. [17] noted that distinct C=O-C peaks and C-O-C peaks formed on the polymer surfaces of a glossy, Vinyl Acetate-based coating [1]. Additionally, the increase in the S=O peak observed at 1200 cm^−1^ suggests the possible existence of hydrogen persulfate residue fibers. These results imply that the Vinyl Acetate modification imparts a hydrophobic character to PP fibers, potentially enhancing their interaction with the cement matrix [18].

**Figure 7:**
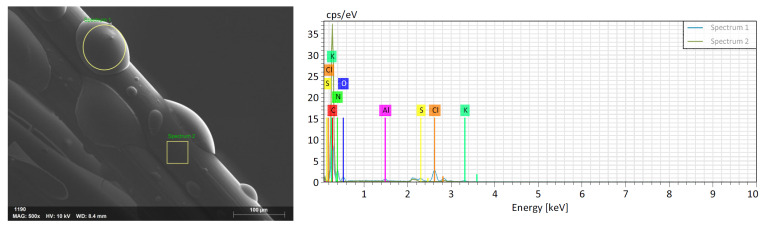
SEM image and EDS graph of PP fiber with Vinyl Acetate.

The SEM image and EDS analysis of the PP fiber with Vinyl Acetate are shown in Figure 7, and the normalized mass concentration (%) values are provided in Table 6.

### 3.2. Cementitious System Properties

#### 3.2.1. Fresh Properties

**Figure 8:**
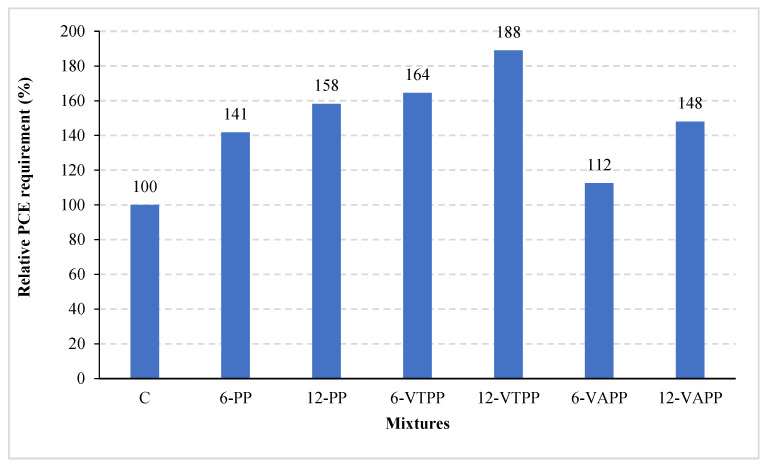
Relative PCE requirement of mixtures.

Figure 8 illustrates the relative amounts of Polycarboxylate Ether (PCE) required for mortar mixtures containing fibers that have been surface-treated with 1-Vinyl-1,2,4-Triazole (VTPP) and Vinyl Acetate (VAPP) to achieve the target flow, in comparison to the control mixture without fibers.

As shown in Figure 8, the addition of fibers to the mixtures resulted in an increased PCE ratio required to achieve the target flow value, regardless of the fiber type and length. Furthermore, the PCE requirement slightly increased with longer fibers, regardless of the fiber type. This behavior is attributed to the fibers enhancing the cohesion of the mixture, which in turn negatively affects workability. Similar findings have been reported in previous studies [4,19,20]. Kaya et al. [4] also noted that the water absorbed onto the fiber surface reduces workability.

**Figure 9:**
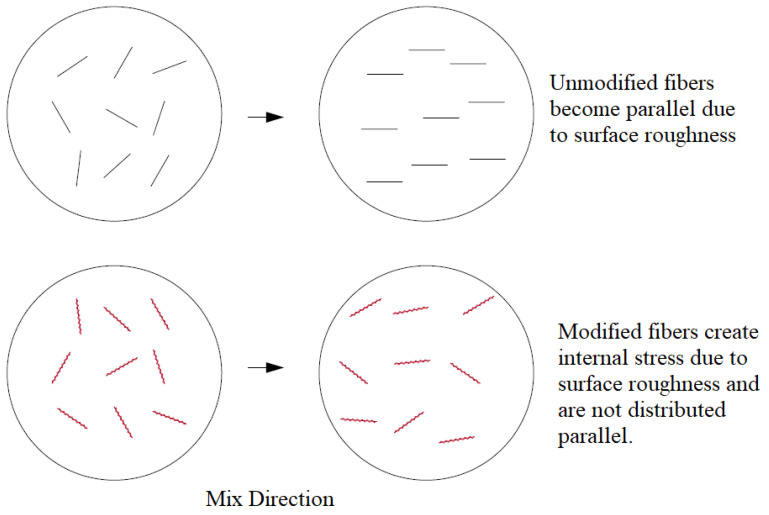
Air entrainment mechanism of roughened surface fibers during mixing [21].

In the case of mixtures containing surface-treated fibers, the PCE requirement increased by approximately 20–30% for VTPP mixtures compared to the standard PP fiber mixtures. 1-Vinyl-1,2,4-Triazole is known for its strong water interaction ability and hydrophilic properties. It is well soluble in water and commonly used in hydrophilic polymer synthesis. When combined with sulfonate-containing compounds (e.g., sodium vinylsulfonate), it forms copolymers that enhance water retention [12]. Therefore, the modification of PP fibers with 1-Vinyl-1,2,4-Triazole likely increases the surface roughness of the fibers, negatively impacting processability by reducing mixing water due to the increased wettability and difficulty dispersing the fibers in the mixture (Figure 9).

In contrast, the VAPP fiber mixtures exhibited an opposite trend. While the PCE requirement increased in VAPP fiber mixtures compared to the non-fiber mixtures, it decreased by 10% compared to the PP fiber mixtures. This behavior can be attributed to the presence of Vinyl Acetate in the VAPP fibers, which imparts hydrophobic properties to the polymer. Hydrophobicity refers to the ability to repel water, and this property is evident on the surface of polymers containing Vinyl Acetate. These polymers are known to quickly regain their hydrophobic characteristics after plasma modification, as confirmed by water contact angle measurements. For instance, polymers with higher Vinyl Acetate content recover their hydrophobic properties more rapidly after plasma treatment [18].

As a result, the water retention on the surface of VAPP fibers is lower than that on unmodified PP fibers. The water that is not adsorbed onto the fiber surface contributes to improved processability by mixing with the free water in the mixture. Therefore, the Vinyl Acetate modification positively influences the processability performance, likely due to the smoother and more hydrophobic surface of the VAPP fibers compared to the VTPP fibers.

#### 3.2.2. Compressive and Flexural Strength Performance

Compressive and flexural strength results of the mixtures are given in Table 7.

As can be seen from Table 7, the strength performance increases with the increase in the curing time in all mixtures. In addition, while the compressive strength is negatively affected by the increase in fiber length in the mixtures, the flexural strength is generally positively affected. It is thought that the decrease in compressive strength is due to the increase in the void ratio in the matrix due to the increase in fiber length. Since the crack bridging mechanism is more dominant in flexural strength, the voids in question did not have much effect. Similar statements have been reported by other researchers [4,11,20].

**Figure 10:**
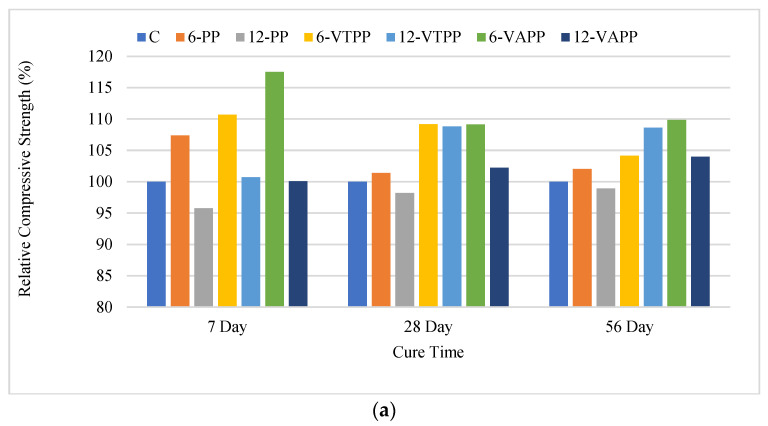

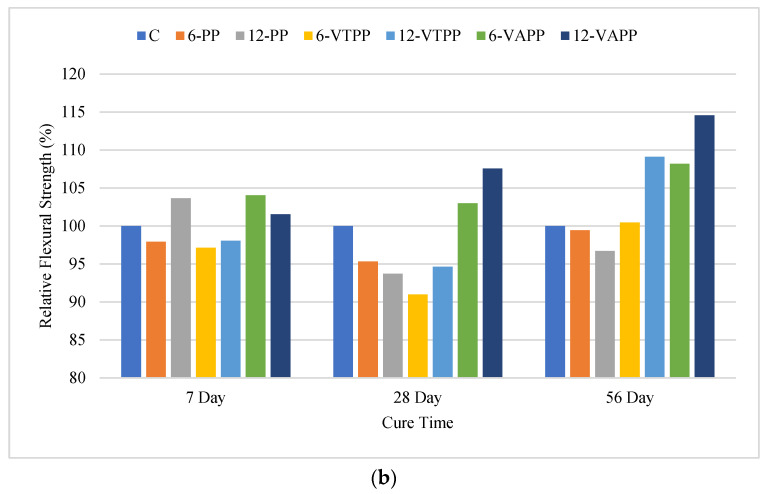
(**a**) Relative compressive strength results of the mixtures. (**b**) Relative flexural strength results of the mixtures.

For a better comparison of compressive and flexural strengths in the presence of fibers, the relative strength results of all mixtures containing fibers compared to the control mixture are given in Figure 10.

When Figure 10a is examined, it is seen that the use of 6 mm fibers (6-PP, 6-VTPP, 6-VAPP), especially in early-age strength, significantly increases the compressive strength compared to the control mixture. This positive effect decreases with the increase in the curing period. This situation is associated with the short fibers contributing to the strength by preventing shrinkage cracks at an early age. The early-age compressive strengths of the mixtures containing 6 mm VTPP and VAPP fibers were higher compared to the control and normal PP fiber mixtures. The mixtures containing 6 mm VTPP and VAPP fibers showed approximately 10 and 17% higher compressive strength performance compared to the control mixture, respectively.

At advanced ages (28 and 56 days), similar mechanisms were observed in mixtures containing 6 mm fibers as in early-age mixtures. However, the strength development was not as high as in 7-day mixtures. While a 1–2% increase was observed in 28- and 56-day mixtures with 6 mm normal PP fibers, there was an increase of 5–10% in 6 mm VTPP and VAPP fiber mixtures.

When 12 mm long fibers were used, the strength of normal PP fiber mixtures decreased compared to the control mixture at early ages, while the compressive strength in 12-VTPP and 12-VAPP fiber mixtures did not change significantly.

When the 28- and 56-day strengths of 12 mm long fiber mixtures were examined, no significant effect of untreated PP fibers was observed in compressive strength compared to the control. Positive effects of 12 mm VTPP and VAPP fibers on 28- and 56-day compressive strength were observed at rates of 5–12%.

It is thought that the performance loss caused by the use of long fibers in mixtures is due to the long fibers clumping together during mixing, entraining air into the mixture and not being able to show their full effect [19].

When all mixtures containing fibers were examined, 6 mm VAPP fibers were more effective than others on the compressive strength of mortar mixtures in advanced compressive strengths. When fiber modification is considered, it is seen that the most efficient modification in terms of compressive strength is the Vinyl Acetate modification. When Figure 10b is examined, it is seen that the increase in fiber length, contrary to compressive strength, generally improves flexural strength. When 7-day flexural strengths are examined, VAPP fiber mixtures showed slightly higher flexural strength performance than the control. The flexural strengths of normal PP and VTPP fiber mixtures decreased slightly except for the 12-VTPP mixture. When advanced flexural strengths (28 and 56 days) are examined, it is seen that VAPP fibers are more effective compared to others.

As is known, fiber prevents the propagation of microcracks formed in the matrix and distributes the load from the weakest region to the stronger regions with the bridging effect [1,4,5,6,14,22]. Thus, it causes an increase in the tensile and flexural strength of the mixtures. In this context, it is seen that the Vinyl Acetate modification is the most successful modification in terms of flexural strength. The addition of fibers to mixtures can have both positive and negative effects in terms of mechanical properties [20]. With the use of rough-surfaced and hydrophilic fibers, air can be entrained into the mixture during mixing, and thus, the strength performance can be negatively affected. In the case where the fibers do not entrain air into the mixture, their performance varies depending on the fiber length. Short fibers such as 6 mm are not sufficient in the bridging function in the matrix and can contribute less to the flexural strength. However, since the fibers in question are more effective on shrinkage cracks, they can make a positive contribution to the compressive strength.

**Figure 11:**
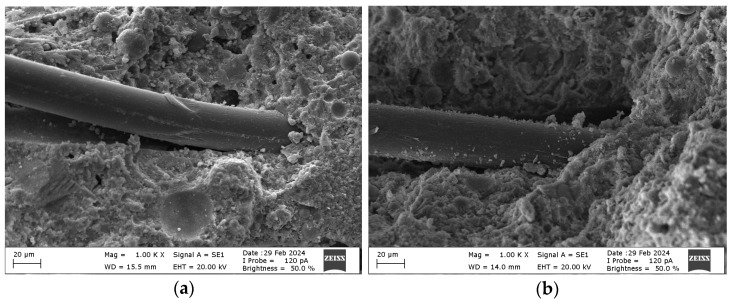
(**a**) Adherence of unmodified PP fiber. (**b**) Adherence of VAPP fiber.

Among the surface modifications, in a study conducted on the adsorption properties and surface coating effects of 1-Vinyl-1,2,4-Triazole on gold surfaces, it was stated by Meltonyan et al. [23] that this compound increased the surface roughness. In addition, in a study conducted on the synthesis and properties of Poly (1-vinyl-1,2,4-triazole) and silver nanocomposites, it was stated that this polymer had high hydrophilicity and was examined in aqueous solutions [24]. In this study, it is thought that VTPP fiber increases the surface roughness and hydrophilicity thanks to these mechanisms. It is seen that the surface roughness of the fiber positively affects the mechanical properties of mortar mixtures. As can be seen from Figure 11, fiber surface modification has a positive effect on matrix–fiber adhesion. However, the hydrophilic property of VTPP fibers causes the accumulation of water molecules in the matrix–fiber interface region. Thus, it was determined that the VTPP fibers in question weaken the mechanical properties in some cases or cannot fully show the effect of the fiber. The decrease in bending strength is thought to be due to this mechanism.

In a study conducted on the surface properties of polymers containing Vinyl Acetate, Squillace et al. [17] stated that this compound reduces hydrophilicity and surface energy. In a study conducted on the synthesis of poly (ethyleneglycol)-b-poly (vinylacetate) block copolymers in various morphologies, it was stated that Vinyl Acetate increases hydrophobic properties and increases water contact angle [25]. In this context, it is thought that Vinyl Acetate reduces the hydrophilicity of VAPP fibers and increases the performance of VAPP fibers on mechanical properties in mortar mixtures due to providing hydrophobic properties to the fiber. Thanks to the hydrophobic properties of VAPP fibers, they can reduce the formation of voids due to the lack of water at the matrix–fiber interface. In this way, it is thought that it improves mechanical properties due to the stronger matrix–fiber adhesion.

## 4. Conclusions

In this study, where the performance of recycled polypropylene fibers in cementitious systems was examined with different modifications, the following results were obtained:

- With the addition of fibers to the mixture, the PCE requirement for the target flow value increased by 12–88% regardless of the fiber type and length.
- Among the modifications applied to recycled waste PP fibers, it was observed that the modification with Vinyl Acetate showed superior performance in terms of flow performance compared to the modification with 1-Vinyl-1,2,4-Triazole.
- Regardless of the fiber type, the compressive strength performance of the mixtures was negatively affected in the range of 4–10% with the increase in fiber length. This situation is thought to be due to the air entrainment of long fibers during mixing.
- Regardless of the modification type, the compressive strength was positively affected in the range of 2–10% by modifying PP fibers.
- It was determined that the modifications were more effective on the flexural strength rather than the compressive strength. This situation shows that the fiber–matrix adhesion was significantly strengthened with the modification.
- As a result, it was observed that fiber–matrix adhesion enhancement can be provided to fibers with poor surface roughness, especially in terms of the flexural strength performance of concrete, with various modifications. Therefore, in future studies, performance increases should be examined by applying different modification methods to different fiber types.

## Figures and Tables

**Table 1:** Chemical composition and physical and mechanical properties of cement.

Oxide (%)	Cement	Physical Properties		
SiO_2_	18.86	Spesific gravity C_2_S (%) C_3_A (%) C_4_AF (%)		3.15
Al_2_O_3_	5.71	Mechanical properties		
Fe_2_O_3_	3.09	Compressive strength (MPa)	1-day	14.7
CaO	62.70		2-day	26.80
MgO	1.16		7-day	49.80
SO_3_	2.39		28-day	58.5
Na_2_O + 0.658 K_2_O	0.92	Fineness		
Cl^−^	0.01	Specific surface (Blaine, cm^2^/g)		3530
Insoluble residue	0.32	Residue on 0.045 mm sieve (%)		7.6
Loss of ignition	3.20			
Free CaO	1.26			

**Table 2:** Key properties of the water-reducing admixture.

Type	Density (gr/cm^3^)	Solid Content (%)	pH	Chloride Content (%)	Alkaline Content Na_2_O (%)
PCE	1.097	36.35	3.82	<0.1	<10

**Table 3:** Mechanical and physical properties of fibers.

Raw Material	Density (gr/cm^3^)	Length (mm)	Tensile Strength (N/mm^2^)	Modulus of Elasticity (N/mm^2^)	Melting Point (°C)
PP	0.91	6–12	450–700	3000–3500	162

**Table 4:** Material quantities required for the production of a 1 dm3 mortar mix (g/dm^3^).

Mixture Code	Cement (g/dm^3^)	Water (g/dm^3^)	Sand (g/dm^3^)	PCE (g/dm^3^)	Fiber (g/dm^3^)	Flow (mm)
C	550	266.75	1512.50	1.27	-	182
6-PP	550	266.75	1499.05	1.80	4.550	205
12-PP	550	266.75	1499.05	2.01	4.550	181
6-VTPP	550	266.75	1499.05	2.09	4.550	180
12-VTPP	550	266.75	1499.05	2.40	4.550	202
6-VAPP	550	266.75	1499.05	1.43	4.550	182
12-VAPP	550	266.75	1499.05	1.88	4.550	180

**Table 5:** Normalized mass concentration (%) table for PP fiber with 1-Vinyl-1,2,4-Triazole.

Spektrum	Carbon	Nitrogen	Oxygen	Sodium	Chlorine	Potassium
Spectrum 1	92.91	0.00	4.28	0.18	1.32	0.27
Spectrum 2	65.45	10.90	11.62	0.76	4.83	0.75

**Table 6:** Normalized mass concentration (%) table for PP fiber with Vinyl Acetate.

Spectrum	Carbon	Nitrogen	Oxygen	Sodium	Chlorine	Potassium
Spectrum 1	46.57	31.00	8.13	1.97	10.14	1.47
Spectrum 2	91.71	6.99	0.54	0.21	0.42	0.00

**Table 7:** Compressive and flexural strength results of the mixtures.

	Compressive Strength (MPa)			Flexural Strength (MPa)		
	7 Day	28 Day	56 Day	7 Day	28 Day	56 Day
C	39.6	50.54	54.09	7.72	8.76	8.79
6-PP	42.53	51.24	55.2	7.56	8.35	8.74
12-PP	37.92	49.63	53.5	8.0	8.21	8.5
6-VTPP	43.83	55.17	56.34	7.5	7.97	8.83
12-VTPP	39.89	54.99	58.76	7.57	8.29	9.59
6-VAPP	46.53	55.16	59.41	8.13	9.02	8.51
12-VAPP	39.63	51.66	56.24	7.34	8.32	10.07

## Data Availability

The original contributions presented in this study are included in the article. Further inquiries can be directed to the corresponding author.
